# The prognostic predictive value of the components of the PR interval in hospitalized patients with heart failure

**DOI:** 10.1186/s12872-022-03028-3

**Published:** 2023-03-08

**Authors:** Yi-Wen Yu, Yan Huang, Xue-Mei Zhao, Lang Zhao, Peng-Chao Tian, Qiong Zhou, Mei Zhai, Yun-Hong Wang, Yu-Hui Zhang, Jian Zhang

**Affiliations:** State Key Laboratory of Cardiovascular Disease, Heart Failure Center, Fuwai Hospital, National Center for Cardiovascular Diseases, Chinese Academy of Medical Sciences and Peking Union Medical College, No. 167 North Lishi Road, Xicheng District, Beijing, China

**Keywords:** Heart failure, P wave duration, PR segment, Prognosis

## Abstract

**Objective:**

Previous reports on the epidemiology, influencing factors, and the prognostic value of the components of PR interval in hospitalized heart failure patients were limited.

**Methods:**

This study retrospectively enrolled 1182 patients hospitalized with heart failure from 2014 to 2017. Multiple linear regression analysis was used to explore the association between the components of PR interval and the baseline parameters. The primary outcome was all-cause death or heart transplantation. Multivariable-adjusted Cox proportional hazard regression models were constructed to explore the predictive value of the components of PR interval for the primary outcome.

**Results:**

In multiple linear regression analysis, higher height (for every 10 cm increase in height: regression coefficient 4.83, P < 0.001) as well as larger atrial and ventricular size were associated with larger P wave duration but not with PR segment. The primary outcome occurred in 310 patients after an average follow-up of 2.39 years. Cox regression analyses revealed that the increase in PR segment was an independent predictor of the primary outcome (every 10 ms increase: hazard ratio 1.041, 95% confidence interval [CI] 1.010–1.083, P = 0.023), whereas the P wave duration did not show significant correlation. When adding the PR segment to an initial prognostic prediction model, the likelihood ratio test and categorical net reclassification index (NRI) showed a significant improvement, but the increase in C-index was not significant. In subgroup analysis, increased PR segment was an independent predictor of the primary endpoint in patients taller than 170 cm (each 10 ms increase: hazard ratio 1.153, 95% CI 1.085–1.225, P < 0.001) but not the shorter group (P for interaction = 0.006).

**Conclusions:**

In hospitalized patients with heart failure, longer PR segment was an independent predictor of the composite endpoint of all-cause death and heart transplantation, especially in the taller group, but it had limited significance in improving the prognostic risk stratification of this population.

**Supplementary Information:**

The online version contains supplementary material available at 10.1186/s12872-022-03028-3.

## Introduction

The global burden of heart failure is still heavy and will increase in the foreseeable future [1]. Heart failure patients can show a variety of cardiac structural and functional abnormalities. Based on the pathological state, the characteristics and prognostic value of some electrocardiographic indicators in this population may be different from those in the community and non-heart failure patients.

It is generally believed that the PR interval on the electrocardiogram reflects the duration from the generation of the electrical activity in the sinus node and through its conduction in the internodal tract, the atrioventricular node, the His bundle, and the Purkinje fiber. The prognostic value of the PR interval was controversial in the community population [2–5], patients with stage A or stage B heart failure, patients who met the indications for cardiac resynchronization therapy [6–11], as well as hospitalized heart failure patients [12]. We wondered if the inconsistency was due to different prognostic values of the two components of the PR interval, i.e. the P wave duration and the PR segment.

The Copenhagen electrocardiogram (ECG) study [13] found that significant shortening and prolongation of the P wave duration (less than the 5th percentile or greater than the 95th percentile) in the community population were both independent predictors of cardiovascular death. In recent years, P wave duration greater than 120 ms has been defined as interatrial block (IAB), and its prognostic significance in patients with heart failure was inconsistent in different studies [14–16]. The PR segment was not considered an independent predictor of all-cause death in the Third National Health and Nutrition Examination Survey [17], and its prognostic value in the heart failure population has not yet been reported.

In the presented retrospective cohort study, we consecutively enrolled hospitalized heart failure patients in a tertiary center for three years, with a median follow-up of 2.39 years, and aimed to explore the distribution characteristics, influencing factors and prognostic value of the P wave duration and PR segment.

## Methods

### Study population

This study retrospectively enrolled hospitalized patients diagnosed with stage C heart failure from 2014 to 2017. Exclusion criteria: (1) under 18 years old; (2) did not meet the diagnostic criteria of heart failure; (3) absence of body surface 12-lead ECG data; (4) non-sinus rhythm (including atrial fibrillation, atrial flutter, junctional rhythm, and ventricular rhythm), second or third-degree atrioventricular block, or pre-excitation syndrome; patients diagnosed with paroxysmal atrial fibrillation but baseline ECG suggesting sinus rhythm were not excluded; (5) after implantation of a pacemaker, cardiac resynchronization pacemaker, or implantable cardioverter defibrillator; (6) died during the hospitalization.

### Clinical data and transthoracic echocardiography

Patient demographic data, New York Heart Association (NYHA) functional class, physical examination at admission, comorbidities, drug use, and laboratory results were collected by qualified cardiologists by reviewing previous medical records. The estimated glomerular filtration rate was calculated according to the modified MDRD formula [18]. The diagnosis of heart failure was determined by at least two cardiologists according to the latest guidelines [19, 20] at the time of admission. Stage C heart failure was defined as structural heart disease with prior or current symptoms of heart failure [19]. For all included patients, transthoracic echocardiographic indicators including wall thickness and atrioventricular diameters were collected and measured by qualified sonographers in Fuwai Hospital according to the latest guidelines [21]. The left ventricular end-systolic and end-diastolic diameters were measured by the biplane Simpson’s method, and the left ventricular ejection fraction (LVEF) was calculated accordingly.

### Twelve-lead ECG data collection

The standard body surface 12-lead electrocardiogram of the selected patients was collected at admission. Three models of twelve-lead electrocardiograph were used for ECG acquisition: Fukuda FX7402, Fukuda FX8322 (Fukuda Corporation, Tokyo, Japan), and GE MAC1200ST (GE Medical Systems *Information Technologies*, the United States). The participant was required to rest quietly for 5 min in a supine position. Then a standard 12-lead ECG was collected for 10 s at a paper speed of 25 mm/s and a voltage of 1 mV for every 10 mm amplitude. Among the ECG parameters, heart rate, PR interval, P wave duration, QRS complex duration, QT interval, and corrected QT interval were automatically measured by the electrocardiographs. For those who did not report the above data due to the acquisition mode, we used the distance tool of Foxit PDF Reader software to manually measure the required data on lead II in the pdf format of the ECG image.

Taking into account the thickness of the ECG waveform line, the distance of segments was measured from the left edge of the starting point to the left edge of the endpoint. The measurement of each segment was performed independently by two qualified cardiologists (YY and XZ) who were blinded to the patients' clinical status and prognosis. If the interpretation result was uncertain, it would be decided after a discussion with senior cardiologists. The manually measured QT interval was corrected by heart rate according to the Bazett’s formula [22]. Interobserver agreement was assessed by comparing the P wave duration measurements of the two observers (YY and XZ) in 50 randomly selected patients. Intraobserver agreement was calculated by repeated measurements in 50 patients by the two observers 1 month after the initial measurement.

Among the research variables, the PR interval was defined as the duration from the time the P wave leaves the baseline to the time the next QRS complex leaves the baseline. The duration of the P wave was defined as the time from the start to the end of the P wave. The PR segment was defined as the time from the end of the P wave to the time the first QRS complex leaves the baseline, which can be calculated from the time difference between the PR interval and the P wave duration.

### Follow-up and outcome

The primary endpoint of this study was defined as the composite of all-cause death or heart transplantation, whichever occurred earlier was recognized as the outcome event. Regular telephone or outpatient follow-ups were conducted to find out the outcome status. Outcome determination was implemented by qualified cardiologists who had undergone standard training.

The research protocol strictly complied with the Declaration of Helsinki and has been approved by the Ethics Committee of Fuwai Hospital affiliated to Peking Union Medical College. All participants have signed written informed consent.

### Statistical methods

The normal distributed continuous variables were represented by the mean (standard deviation). The non-normal distributed continuous variables were represented by the median, the 25th percentile, and the 75th percentile. The categorical variables were represented by the frequency (percentage). For the baseline data grouped by binary categorical variables, the normally distributed continuous variables, non-normally distributed continuous variables, and categorical variables were compared using the two independent sample t-test, Mann–Whitney U test, chi-square test or Fisher's exact test, respectively.

In the univariable correlation analysis between baseline variables and the research variables, Pearson correlation analysis was used for binormal continuous variables, point-biserial correlation analysis was used for binary variables and continuous variables, and Spearman rank correlation was used for other cases. For the baseline variables that are significantly correlated with the research variables in the single-factor correlation analysis, the stepwise regression method was used to construct a multiple linear regression model to explore the possible influencing factors of the research variables. The multiple imputation method was used to deal with the missing values ​​in the original data.

In this study, the Kaplan–Meier method was used to draw the unadjusted survival curves, and the log-rank test was used for comparison between groups. Unadjusted, age and gender-adjusted, and multivariable-adjusted Cox proportional hazard regression models were constructed in the original cohort to explore the predictive value of the research variables for the primary outcome. The combinations of covariates in the multivariable-adjusted Cox model were determined based on the significant variables in the univariable Cox regression combined with clinical experience. The multivariable-adjusted Cox regression model 1 was adjusted for age, sex, body mass index, systolic blood pressure, natural log-transformed N-terminal pro-B-type natriuretic peptide (NT-proBNP) level, estimated glomerular filtration rate, heart rate, QRS complex duration, and the use of angiotensin-converting enzyme inhibitors/angiotensin receptor blockers (ACEI/ARBs). The multivariable-adjusted Cox regression model 2 was adjusted for age, sex, NYHA class, natural log-transformed NT-proBNP level, platelet count, serum chloride, serum creatinine, and serum uric acid levels. The multivariable Cox regression model 3 was adjusted for age, sex, NYHA class, natural log-transformed NT-proBNP level, the use of digoxin, ACEI/ARBs, aldosterone receptor antagonists, and the use of diuretics.

As the sensitivity analysis, we constructed a propensity score-matched cohort and performed multivariable-adjusted Cox proportional hazard regression analysis in this population. The propensity score was calculated by multivariable logistic regression analysis. The matching variables of the propensity score-matched cohort included age, sex, systolic blood pressure, history of hypertension, use of ACEI/ARBs, hemoglobin level, serum albumin level, serum potassium level, estimated glomerular filtration rate, heart rate, and QRS complex duration. The multivariable Cox regression model in the propensity score-matched cohort was adjusted for covariates the same as model 1 in the original cohort. The propensity score-matched cohort was matched at a ratio of 1:1 by the nearest neighbor matching method without replacement, and the balance diagnosis was evaluated by calculating the standardized mean differences of the baseline variables after matching.

We used the likelihood ratio test, categorical and continuous net reclassification index [23] (NRI), the integrated discrimination improvement index [24] (IDI), and the concordance index (C-index) to evaluate the additional predictive value for the composite endpoint of all-cause death or heart transplantation after the PR segment is added to the initial prediction model (including age, systolic blood pressure, estimated glomerular filtration rate, and peripheral blood NT-proBNP level).

In addition, the interaction between research variables and covariates was explored in the multivariable Cox proportional hazard regression model, and subgroup analysis was performed accordingly.

A two-sided P < 0.05 was defined as statistically significant. All statistical analysis was performed using R software version 3.6.2 (R Foundation for Statistical Computing).

## Results

### Baseline characteristics

A total of 1182 hospitalized heart failure patients were finally included, of which 894 (75.6%) were male. The average age and standard deviation were 54.35 ± 16.08 years old. The median (25th–75th percentiles) was 114.00 ms (104.00–126.00 ms) for P wave duration, and 57.97 ms (42.00–75.36 ms) for PR segment, respectively.

Table 1 shows the baseline characteristics of the total cohort grouped by the primary endpoint. After an average follow-up of 2.39 years (interquartile range 0.77 to 3.48 years), 310 out of the 1182 patients experienced the primary outcome.

**Table 1:** Baseline characteristics of the total population grouped by the primary outcome events

	Total (n = 1182)	No events (n = 872)	Events (n = 310)	*P* value
Age (years)	54.35 (16.08)	52.87 (15.70)	58.51 (16.43)	< 0.001
Sex				0.996
Male	894 (75.6)	659 (75.6)	235 (75.8)	
Female	288 (24.4)	213 (24.4)	75 (24.2)	
Height (cm)^a^	169.05 (7.93)	169.26 (7.84)	168.45 (8.18)	0.129
Weight (kg)^a^	72.68 (15.98)	74.23 (15.98)	68.22 (15.13)	< 0.001
BMI (kg/m^2^)^a^	25.22 (4.52)	25.70 (4.46)	23.85 (4.41)	< 0.001
SBP (mmHg)	121.29 (21.38)	123.42 (20.84)	115.30 (21.76)	< 0.001
DBP (mmHg)^a^	73.20 (14.00)	74.40 (14.20)	69.82 (12.87)	< 0.001
NYHA class^a^				< 0.001
I	55 (5.3)	52 (6.9)	3 (1.1)	
II	277 (26.8)	244 (32.1)	33 (12.1)	
III	509 (49.3)	370 (48.7)	139 (50.9)	
IV	191 (18.5)	93 (12.3)	98 (35.9)	
LVEF (%)^a^	36.00 [28.00, 50.00]	38.00 [30.00, 53.00]	31.50 [25.00, 45.00]	< 0.001
HF types				< 0.001
HFrEF	719 (60.8)	499 (57.2)	220 (71.0)	
Non-HFrEF	463 (39.2)	373 (42.8)	90 (29.0)	
Comorbidities				
Hypertension	624 (52.8)	478 (54.8)	146 (47.1)	0.023
Diabetes	368 (31.1)	257 (29.5)	111 (35.8)	0.046
Coronary artery disease	509 (43.1)	369 (42.3)	140 (45.2)	0.423
Myocardial infarction	454 (38.4)	322 (36.9)	132 (42.6)	0.091
Dilated cardiomyopathy	309 (26.1)	225 (25.8)	84 (27.1)	0.711
Hypertrophic cardiomyopathy	19 (1.6)	15 (1.7)	4 (1.3)	0.799
Laboratory results				
NT-proBNP (pg/ml)^a^	1977.00 [648.50, 5273.50]	1448.50 [469.25, 3948.00]	4063.00 [1821.00, 9685.00]	< 0.001
ln transformed NT-proBNP^a^	7.43 (1.56)	7.14 (1.57)	8.26 (1.19)	< 0.001
Hemoglobin (g/L)^a^	139.66 (22.27)	141.10 (21.94)	135.61 (22.71)	< 0.001
Platelet count (× 10^9^/L)^a^	221.36 (73.16)	226.63 (74.33)	206.59 (67.74)	< 0.001
Albumin (g/L)^a^	39.83 (5.37)	40.44 (5.14)	38.15 (5.65)	< 0.001
Potassium (mmol/L)^a^	4.00 (0.49)	3.98 (0.47)	4.06 (0.54)	0.008
Sodium (mmol/L)^a^	138.18 (3.85)	138.70 (3.39)	136.71 (4.61)	< 0.001
eGFR (ml/min/1.73m^2^)^a^	72.98 [56.94, 88.96]	76.79 [61.27, 90.84]	61.34 [46.07, 77.27]	< 0.001
Uric acid (µmol/L)^a^	457.23 [351.68, 572.97]	441.38 [338.67, 561.02]	503.74 [396.97, 616.21]	< 0.001
12-lead ECG parameters				
Heart rate (bpm)	78.53 (15.69)	78.10 (15.54)	79.75 (16.06)	0.111
QRS complex duration (ms)	109.65 (26.01)	107.13 (24.23)	116.74 (29.36)	< 0.001
QT interval (ms)	406.31 (48.51)	405.45 (45.96)	408.73 (55.04)	0.307
QTc (ms)	458.67 (44.27)	456.92 (43.38)	463.58 (46.39)	0.023
PR interval (ms)	170.00 [156.52, 192.00]	168.12 [156.00, 189.84]	177.00 [160.00, 200.00]	< 0.001
P wave duration (ms)	114.00 [104.00, 126.00]	114.00 [104.00, 126.00]	115.94 [104.00, 127.88]	0.227
PR segment (ms)	57.97 [42.00, 75.36]	56.00 [40.58, 72.00]	62.00 [46.38, 84.92]	< 0.001
Echocardiography parameters				
LAD (mm)^a^	42.73 (7.30)	42.01 (7.07)	44.76 (7.57)	< 0.001
LVEDD (mm)^a^	61.96 (12.01)	61.13 (11.32)	64.26 (13.53)	< 0.001
IVS (mm)^a^	9.85 (2.42)	9.85 (2.32)	9.83 (2.70)	0.867
LVPW (mm)^a^	9.45 (1.74)	9.45 (1.62)	9.44 (2.07)	0.941
RVD (mm)^a^	24.03 (5.04)	23.82 (4.89)	24.64 (5.42)	0.016
Medication				
Digoxin^a^	530 (45.7)	383 (44.3)	147 (49.7)	0.128
ACEI/ARB^a^	711 (61.3)	584 (67.6)	127 (42.9)	< 0.001
β receptor blockers^a^	1061 (91.5)	795 (92.0)	266 (89.9)	0.307
MRA^a^	829 (71.5)	607 (70.3)	222 (75.0)	0.137
Diuretics^a^	979 (86.9)	725 (87.5)	254 (85.5)	0.454

The normally distributed continuous variables were expressed as the mean (standard deviation), non-normally distributed continuous variables were expressed as the median [25% and 75% percentiles], and categorical variables were expressed as the frequency (percentage). For comparison between groups, the two independent samples t test was used for normally distributed continuous variables, the Krukal–Wallis test was used for continuous non-normally distributed continuous variables, and the chi-square test was used for categorical variables

*ACEI/ARB* angiotensin converting enzyme inhibitor/angiotensin receptor blocker, *BMI* body mass index, *DBP* diastolic blood pressure, *ECG* electrocardiogram, *eGFR* estimated glomerular filtration rate, *HFrEF* heart failure with reduced ejection fraction, *IVS* interventricular septal thickness, *LAD* left atrial dimension, *LVEDD* left ventricular end-diastolic dimension, *LVEF* left ventricular ejection fraction, *LVPW* left ventricular posterior wall thickness, *MRA* mineralocorticoid receptor antagonists, *NT-proBNP* N-terminal pro-B-type natriuretic peptide, *NYHA* New York Heart Association, *RVD* right ventricular dimension, *SBP* systolic blood pressure

^a^Variables with missing data. The numbers of missing values were 50 for height, 98 for weight, 102 for BMI, 1 for DBP, 150 for NYHA class, 1 for LVEF, 143 for NT-proBNP level, 3 for peripheral blood hemoglobin level and platelet count, 8 for LAD, 1 for LVEDD, 35 for IVS and RVD, 37 for LVPW, and 56 for the use of diuretics. There were 22 missing values for the use of digoxin, ACEI/ARB, β receptor blockers, and MRA. There were also 2 missing values for the level of albumin, potassium, sodium, eGFR, and Uric acid

The PR segment was longer in the event group than in the non-event group (62.00 ms compared to 56.00 ms), but the difference in the P wave duration was not significant (115.94 ms compared to 114.00 ms, *P* > 0.05) (Table 1). When grouped by the quartiles of the research variables, the trend test showed that the number of events in each group increases with the increase of the PR segment (P < 0.001), while the number of events in each group of P wave duration did not show any significance (P = 0.24). The above findings suggested that the PR segment may have prognostic predictive value in this population, which is to be verified in the analyses below.

### Correlation analysis of the P wave duration and PR segment with other clinical variables

Several clinical characteristics were significantly related to the P wave duration and the PR segment (Table 2). However, the multiple linear regression model constructed by the above indicators had limited predictive value for the two research variables (coefficient of determination 0.05 and 0.07, respectively). Notably, height was associated with the P wave duration but not with the PR segment.

**Table 2:** Multiple linear regression analysis of the P wave duration and PR segment and the baseline characteristics

		P wave duration (ms) (R^2^ = 0.07)		PR segment (ms) (R^2^ = 0.05)	
		Regression coefficient (standard deviation)	*P* value	Regression coefficient (standard deviation)	*P* value
Age (years)	Per decade increase	0.91 (0.39)	0.020	2.69 (0.55)	< 0.001
Height (cm)	Per 10 cm increase	4.83 (0.72)	< 0.001		
Ln [NT-proBNP]	Per ln [NT-proBNP]	0.76 (0.38)	0.044		
NYHA class	III/IV versus I/II			6.80 (1.89)	< 0.001
HF types	HFrEF versus non-HFrEF	3.71 (1.39)	0.008		
Myocardial infarction	Yes versus no	− 3.45 (1.23)	0.005		
Ln [uric acid]	Per ln [uric acid]			6.19 (2.41)	0.011
Heart rate (bpm)	Per 10 bpm increase			− 1.77 (0.57)	0.002
MRA	Yes versus no	3.50 (1.43)	0.014		

Multiple linear regression was performed in the data after multiple imputation. A negative regression coefficient indicated a negative correlation

*eGFR* estimated glomerular filtration rate, *HF* heart failure, *HFrEF* heart failure with reduced ejection fraction, *MRA* mineralocorticoid receptor antagonists, *NT-proBNP* N-terminal pro-B-type natriuretic peptide, *NYHA* New York Heart Association

As for the association between the research variables and the echocardiography parameters, the P wave duration was weakly correlated with the left atrial anteroposterior diameter, the left ventricular end-diastolic anteroposterior diameter, and the right ventricular anteroposterior diameter measured by transthoracic echocardiography (correlation coefficient 0.15–0.30, P < 0.001). However, the PR segment was scarcely correlated with the sizes of the atria and ventricles (Additional file 1: Fig. S1).

### Survival analysis

**Fig. 1:**
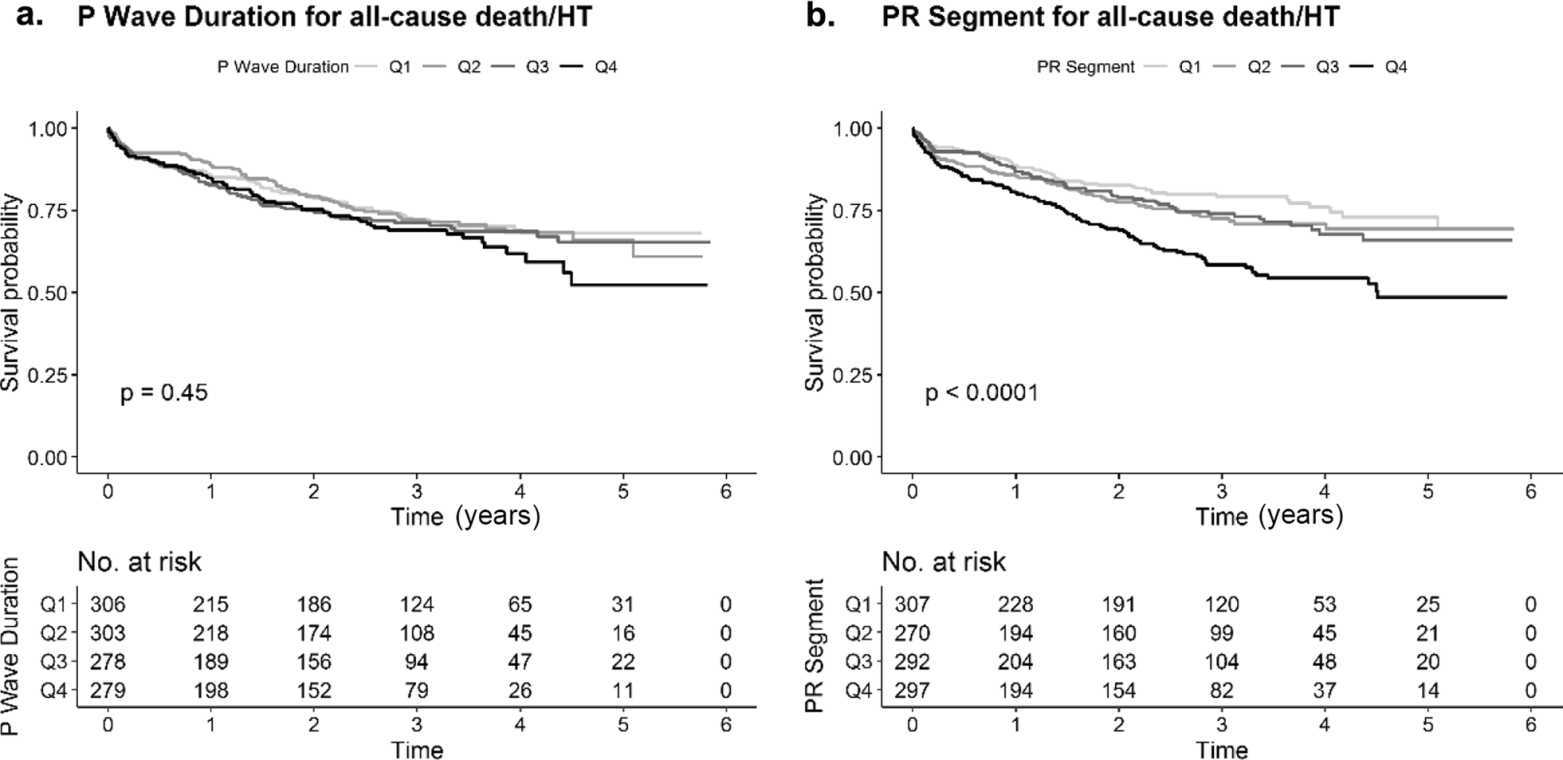
The unadjusted Kaplan–Meier curves for the primary outcome grouped by the quartiles of the research variables. **a** P wave duration; **b** PR segment. *HT* heart transplantation

The unadjusted Kaplan–Meier curve of the primary endpoint grouped by quartiles of the P wave duration and the PR segment was shown in Fig. 1, which implied that the groups with longer PR segments had a higher risk for the primary outcome (log-rank test P < 0.0001), but those with longer P wave duration did not show a significant difference (log-rank test P = 0.45).

There was no significant correlation between the P wave duration and the primary outcome in all uni- and multi-variable Cox regression models (all P > 0.05). As for the PR segment, in the univariable model, the age and sex-adjusted Cox regression model, as well as the multiple Cox regression models adjusted for multiple factors in the original and the propensity score-matched population, the increase of the PR segment was an independent predictor of the primary endpoint (Table 3). The baseline characteristics of the propensity score matched cohort were shown in Additional file 3: Table S1. The balance test of the model was shown in Additional file 2: Fig. S2.

**Table 3:** The predictive value of the P wave duration and the PR segment for the primary outcome

	P wave duration			PR segment		
	Hazard ratio (95% CI)	*P* value	*P* for trend	Hazard ratio (95% CI)	*P* value	*P* for trend
Univariate Cox regression analysis						
Per 10 ms increase	1.030 (0.970, 1.093)	0.335		1.091 (1.055, 1.127)	< 0.001	
Age and sex-adjusted Cox regression analysis			0.115			< 0.001
Per 10 ms increase	1.034 (0.973, 1.099)	0.276		1.072 (1.036, 1.109)	< 0.001	
Q4 versus Q1	1.256 (0.910, 1.734)	0.165		1.923 (1.391, 2.657)	< 0.001	
Multivariable-adjusted Cox regression model 1			0.681			0.015
Per 10 ms increase	0.955 (0.889, 1.020)	0.202		1.041 (1.010, 1.083)	0.023	
Q4 versus Q1	0.869 (0.601, 1.259)	0.459		1.633 (1.118, 2.384)	0.011	
Multivariable-adjusted Cox regression model 2			0.406			0.005
Per 10 ms increase	0.959 (0.897, 1.024)	0.211		1.043 (1.004, 1.083)	0.029	
Q4 versus Q1	0.838 (0.578, 1.216)	0.353		1.734 (1.196, 2.515)	0.004	
Multivariable-adjusted Cox regression model 3			0.891			0.004
Per 10 ms increase	0.983 (0.916, 1.055)	0.633		1.049 (1.010, 1.090)	0.013	
Q4 versus Q1	0.939 (0.642, 1.373)	0.745		1.744 (1.191, 2.554)	0.004	
Multivariable-adjusted Cox regression model in the PSM cohort			0.484			0.005
Per 10 ms increase	0.953 (0.883, 1.029)	0.218		1.076 (1.033, 1.121)	< 0.001	
Q4 versus Q1	0.826 (0.557, 1.225)	0.342		1.854 (1.251, 2.749)	0.002	

Multivariable-adjusted Cox regression model 1 was adjusted for age, sex, body mass index, systolic blood pressure, ln-transformed NT-proBNP, estimated glomerular filtration rate, heart rate, QRS complex duration, and the use of ACEI/ARB. Multivariable-adjusted Cox regression model 2 was adjusted for age, sex, NYHA functional class, ln-transformed NT-proBNP, platelet count, serum chloride level, serum creatinine level, and serum uric acid level. Multivariable-adjusted Cox regression model 3 was adjusted for age, sex, NYHA functional class, ln-transformed NT-proBNP, and the use of digoxin, ACEI/ARB, mineralocorticoid receptor antagonists, and diuretics. The matched covariates in the propensity score matching population included age, sex, systolic blood pressure, a history of hypertension, the use of ACEI/ARB, hemoglobin level, serum albumin level, serum potassium level, estimated glomerular filtration rate, heart rate, and QRS complex duration. The adjusted factors of the multivariable Cox regression model in the propensity score matching cohort were the same as those in model 1 in the original cohort

*ACEI/ARB* angiotensin converting enzyme inhibitor/angiotensin receptor blocker, *NT-proBNP* N-terminal pro-B-type natriuretic peptide, *NYHA* New York Heart Association

### The incremental contribution of the PR segment to risk classification

The evaluation of the incremental contribution of the PR segment to the three-year primary outcome risk was shown in Table 4. Compared with the initial model (including age, systolic blood pressure, estimated glomerular filtration rate, and peripheral blood NT-proBNP level), after adding the PR segment, the reduction of the likelihood score of the prediction model (P = 0.003), the continuous NRI, the IDI, and the categorical NRI results using the three-year primary outcome risk of 10% and 30% as the cut-off values (P = 0.010) all reached statistical significance, but the C-index did not improve significantly.

**Table 4:** Incremental contribution of the PR segment to the existing model for three-year primary outcome prediction

	Likelihood ratio	*P* value for the likelihood ratio test	Categorical NRI (95%CI)	*P* value	Continuous NRI (95%CI)	*P* value	IDI (95%CI)	*P* value	C-index (95%CI)	*P* value
Model 1	− 1577	Reference	–	Reference	–	Reference	–	Reference	0.732 (0.702, 0.763)	Reference
Model 1 + PR segment	− 1572	0.003	0.063 (0.015, 0.110)	0.010	0.148 (0.004, 0.292)	0.045	0.013 (0.005, 0.022)	0.003	0.742 (0.712, 0.772)	0.996

Model 1 included age, systolic blood pressure, estimated glomerular filtration rate, and peripheral blood N-terminal pro-B-type natriuretic peptide level. The cut-off value for the categorical NRI were 10% and 30% for the happening of the primary outcome in three years

*NRI* net reclassification index, *IDI* integrated discrimination improvement index

### Subgroup analysis by sex and height

In the interaction test of the Cox proportional hazard regression analysis in the total population, it was found that sex and height had significant interactions with the prognostic predictive value of the PR segment (P for interaction: sex 0.015 and height 0.006, respectively). The subgroup analysis was performed accordingly (Table 5).

**Table 5:** Multivariable-adjusted Cox proportional hazards regression analysis of the PR segment and the primary outcome grouped by the median height and sex

	PR segment (per 10 ms increase)		PR segment (Q4 versus Q1)		PR segment (Q1–Q4) *P* for trend	Interaction *P* value
	Hazard ratio (95% CI)	*P* value	Hazard ratio (95% CI)	*P* value		
Height						0.006
Height ≤ 170 cm (n = 643)	0.980 (0.931, 1.032)	0.449	0.991 (0.614, 1.600)	0.970	0.769	
Height > 170 cm (n = 489)	1.153 (1.085, 1.225)	< 0.001	3.376 (1.749, 6.516)	< 0.001	< 0.001	
Sex						0.015
Female (n = 288)	0.957 (0.875, 1.046)	0.335	0.884 (0.400, 1.953)	0.761	0.424	
Male (n = 894)	1.075 (1.030, 1.122)	< 0.001	1.986 (1.284, 3.070)	0.002	0.002	
Height & sex						
Female, height ≤ 170 cm (n = 270)	0.955 (0.872, 1.045)	0.317	0.834 (0.374, 1.858)	0.656	0.354	
Male, height ≤ 170 cm (n = 373)	1.002 (0.939, 1.069)	0.958	1.102 (0.597, 2.032)	0.757	0.705	
Male, height > 170 cm (n = 486)	1.152 (1.084, 1.224)	< 0.001	3.338 (1.728, 6.448)	< 0.001	< 0.001	

The multivariable-adjusted Cox regression model was adjusted for age, sex, body mass index, systolic blood pressure, ln-transformed N-terminal pro-B-type natriuretic peptide, estimated glomerular filtration rate, heart rate, QRS complex duration, and the use of angiotensin converting enzyme inhibitor/angiotensin receptor blocker. When testing the interaction, the height was transformed into a binary variable by the median as the cutoff, and the PR segment was transformed into ranked variables grouped by quartiles. The group of the female patients with a height > 170 cm was not displayed in the table because the sample size was only 3

In the male population, the trend test of the research variables grouped by quartiles suggests that the increase in the PR segment was associated with a significant increase in the risk of all-cause death or heart transplantation, and the risk of patients in the fourth quartile was significantly higher than those in the first quantile (P for trend: 0.002). In the female population, the above analysis showed no significant difference (P for trend: all greater than 0.05). When classified by height, the PR segment was significantly associated with the primary outcome in the subgroup taller than the median of the total population (i.e. 170 cm) but not in shorter patients (Table 5).

Furthermore, we made a four-grid table classified by sex and the median of height and found that the sample size of shorter men, taller men, shorter women, and taller women were 373, 486, 270, and 3, respectively. Subgroup analysis was performed in the former three groups (Table 5). It was found that the increase of the PR segment was associated with a significantly increased risk of all-cause death or heart transplantation only in the subgroup of taller men, while in the shorter groups of both men and women, the PR interval and the PR segment were not significantly related to the primary outcome.

### Interobserver and intraobserver variability

The measurement of ECG segments shows high repeatability and reproducibility in our cohort. The intraclass correlation coefficient for interobserver agreement was 0.983 (95%CI: 0.959 to 0.992), and the intraclass correlation coefficient for intraobserver agreement was 0.993 (95%CI: 0.988 to 0.996).

## Discussions

The main findings of this study were as follows: (1) Longer P-wave duration but not PR segment was associated with higher height and larger atrioventricular diameters reported by echocardiography. (2) Longer PR segment was an independent predictor of the primary outcome, while the P wave duration did not show a significant correlation with the primary outcome; whereas adding the PR segment to the existing prognostic prediction model for patients with heart failure improved the predictive accuracy significantly but weakly. (3) Larger PR segment was an independent predictor of an increased risk of the primary outcome in patients taller than 170 cm but not in shorter patients.

The median PR interval in our population was similar to that in the heart failure population reported by Nikolaidou et al. [12]. Compared with reports in a community cohort [17], the mean value of the PR interval, P wave duration, and PR segment was larger in our cohort, which was consistent with the clinical finding that heart failure patients suffered from more severe atrial enlargement and conduction system abnormalities compared to the community population.

We found in this study that the P wave duration but not the PR segment was significantly correlated with height as well as the sizes of atria and ventricles measured by transthoracic echocardiography, which was consistent with previous studies [25–28]. Besides, previous studies have reported the correlations between height and the chamber diameters, suggesting that the correlation between height and P wave duration may be related to its correlation with the chamber diameters, especially the atrium size.

Several studies had reported little prognostic value of the P wave duration in patients with stage C chronic heart failure. Escobar-Robledo et al. [15] reported in a cohort of patients referred to a multidisciplinary heart failure clinic that in patients with P wave duration not less than 120 ms, no matter whether combined with biphasic P wave in the inferior leads, prolonged P wave duration was not an independent predictor of all-cause death. Shturman et al. [29] reported similar results in post-acute myocardial infarction patients. The findings above were in consistent with the results of our study.

To our knowledge, this study is the first to report the prognostic value of the PR segment for hospitalized patients with chronic heart failure. Previous reports in the community population suggested that the PR segment was not an independent predictor of all-cause death [17] or new-onset atrial fibrillation [30]. However, compared with non-heart failure patients, pathological factors such as ischemia and fibrosis that affected the subatrial conduction system in patients with heart failure have greatly increased, thus affecting the disease course and patient prognosis. The prognostic value of the PR segment could be a reflection of the accompanying prognostic factors. Electrophysiological studies in animal models and prospective cohort studies may provide clues for related electrophysiological mechanisms.

Adding the PR segment to the existing prognostic prediction model for patients with heart failure improved the predictive accuracy significantly but weakly. Patients with heart failure usually have heterogeneous underlying diseases, multiple complications, and complex prognostic factors. A single indicator requires a strong prognostic value to significantly improve the existing prognosis prediction models. In previous heart failure prognosis prediction models such as the Seattle heart failure model [31], GISSI-HF model [32], and MAGGIC model [33], only GISSI-HF included ECG parameters (the QRS complex duration and heart rate). However, in confirmatory studies [34, 35], the prognostic value of the models above was not yet satisfactory at individual levels.

In the big data era, the value of ECG is being further unlocked with the methods of artificial intelligence (AI), especially deep learning. The advantages of ECG such as convenience, inexpensiveness, easy to store and transfer into digital format, and containing huge objective information made it a highly appropriate material for the application of novel data mining tools. For deep learning, the feature extraction process was automated, thus avoiding possible bias during the pattern recognition process by human. The ECG already showed promising ability in the prediction of left ventricular systolic dysfunction [36], atrial fibrillation [37], and hypertrophic cardiomyopathy [38] in recent studies. However, the concerns of the deep learning method such as external validity and the incremental benefit on the basis of existing medical practice stay to be addressed in the use of AI models based on ECG.

The modifying effect of height on the prognostic value of the PR segment has not been reported in the same population or outcome as ours. Height, as a seemingly harmless physical examination index, was found to be related to the risk of new-onset atrial fibrillation in the community populations and patients with left ventricular dysfunction in a series of studies [39–42], and the results were consistent in different sexes. The mechanism hypothesis could be the possible genetic correlation between height and atrial fibrillation [43], or larger atrium size in taller patients [44–47], which might increase the risk of new or recurrent atrial fibrillation [48–51], thus exacerbating the cardiovascular prognosis. Whether the enhanced risk of taller patients found in our study was related to the correlation of height with the risk of new-onset atrial fibrillation is to be clarified in the future.

The strength of this study relies on the rigorous statistical analysis and multi-layer authentication of the results. In the survival analysis, multivariate Cox proportional hazard regression models were constructed combining clinical experience rather than only based on the univariate Cox regression results to determine possible confounding factors that need to be adjusted, including strong prognosis predictive factors such as NT-proBNP. The three multivariate Cox models were adjusted respectively for other ECG indicators, laboratory results, and medications in addition to the demographic data. Furthermore, the multivariate Cox model after propensity score matching was used as a sensitivity analysis to minimize the selection bias and to confirm the reliability of the prognostic analysis results.

Its limitation firstly derives from the nature of single-center retrospective cohort study. The confounding factors cannot be adjusted completely, and we could only report the correlation but not causality. The study enrolled only Chinese patients and lacked ethnic variation. Secondly, the external validity of the results also remains to be verified. Thirdly, the cause of death was available for not all the deceased subjects in the current study. Moreover, due to the good compliance with the optimized drug treatment in our cohort, the use of drugs affecting the atrioventricular node electricity conduction such as β-blockers was quite common (91.5%), so the subgroup analysis of the population of patients not using antiarrhythmic drugs was not carried out. Finally, the study adopted a single standard 12-lead electrocardiogram at admission, whereas the ECG of patients with heart failure may change significantly at different stages of the disease course. Therefore, future studies are needed to determine the change of the research variables at admission and discharge or before and after a fixed follow-up period on the prognosis of patients with heart failure.

## Conclusions

In hospitalized patients with heart failure, increased PR segment was an independent predictor of the composite endpoint of all-cause death and heart transplantation, but the incremental prognostic risk stratification value was limited. In the interaction and subgroup analysis, increased PR segment was an independent predictor of enhanced risk for the primary outcome in patients taller than 170 cm but not in shorter individuals.

## Supplementary Information

**Additional file 1. Fig. S1**: Univariable correlation analysis of the P wave duration, the PR segment and other ECG and transthoracic echocardiographic parameters.

**Additional file 2. Fig. S2**: The distribution of the standardized mean differences before and after propensity score matching. A. P wave duration; B. PR segment.

**Additional file 3. Table S1**: Baseline characteristics of the propensity score-matched cohort grouped by the medians of the P wave duration and the PR segment.

## Data Availability

The datasets used or analysed during the current study are available from the corresponding author on reasonable request.

## Abbreviations

ACEI/ARBs: Angiotensin-converting enzyme inhibitors/angiotensin receptor blockers
AI: Artificial intelligence
C-index: Concordance index
ECG: Electrocardiogram
IAB: Interatrial block
IDI: Integrated discrimination improvement index
LVEF: Left ventricular ejection fraction
NRI: Net reclassification index
NT-proBNP: N-terminal pro-B-type natriuretic peptide
NYHA: New York Heart Association
