# Supplementary figures and images for: The prognostic predictive value of the components of the PR interval in hospitalized patients with heart failure
Source: BMC Cardiovasc Disord. 2023 Mar 8;23:119. doi: 10.1186/s12872-022-03028-3 (PMC9996982; doi:10.1186/s12872-022-03028-3)

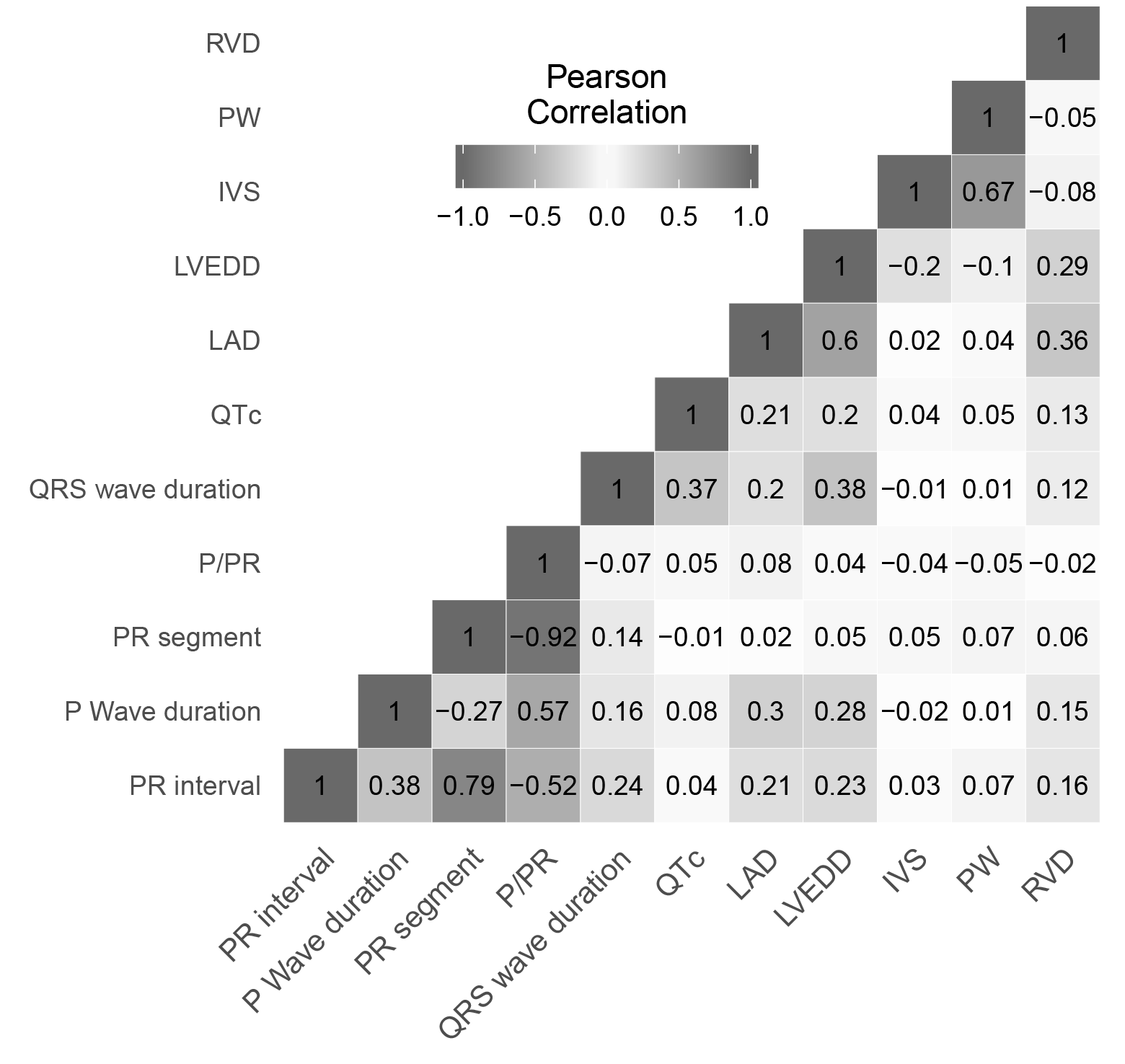

Supplement: Supplementary file 1 — Additional file 1. Fig. S1: Univariable correlation analysis of the P wave duration, the PR segment and other ECG and transthoracic echocardiographic parameters. [file 12872_2022_3028_MOESM1_ESM.tif]

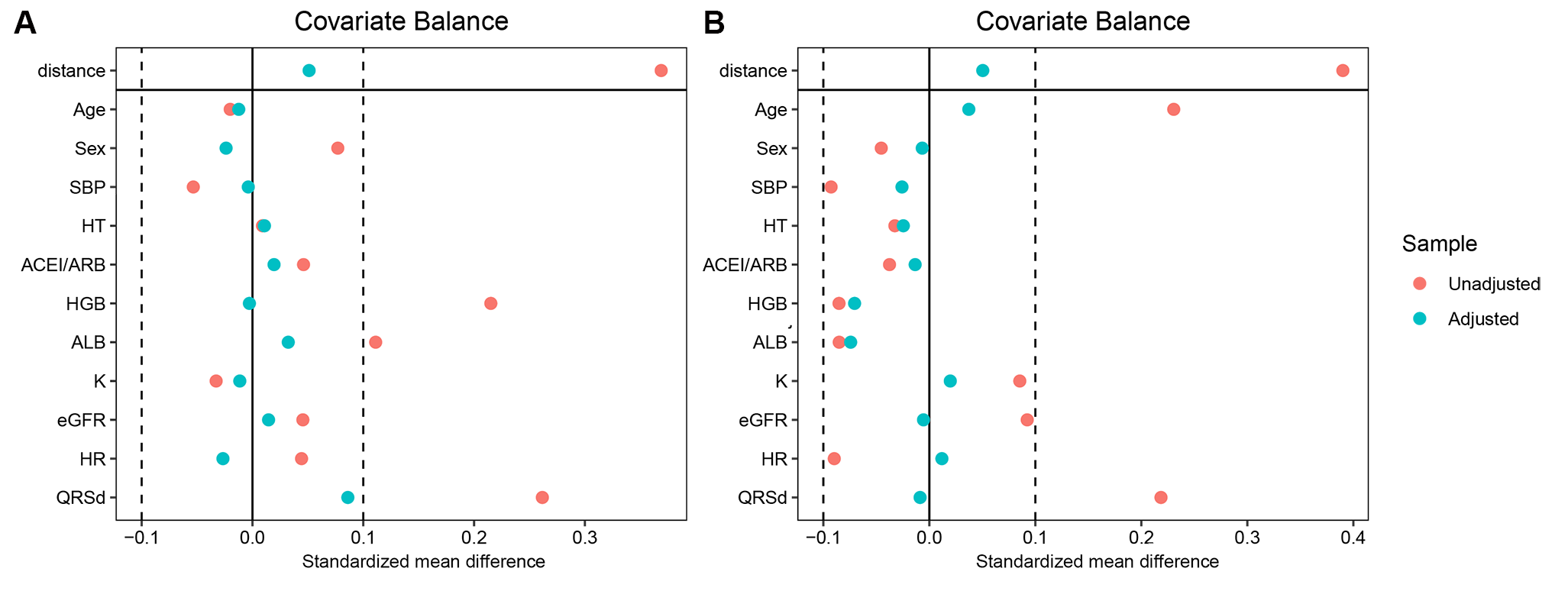

Supplement: Supplementary file 2 — Additional file 2. Fig. S2: The distribution of the standardized mean differences before and after propensity score matching. A. P wave duration; B. PR segment. [file 12872_2022_3028_MOESM2_ESM.tif]
