# Supplementary material for: The prognostic predictive value of the components of the PR interval in hospitalized patients with heart failure
Source: BMC Cardiovasc Disord. 2023 Mar 8;23:119. doi: 10.1186/s12872-022-03028-3 (PMC9996982; doi:10.1186/s12872-022-03028-3)
Supplement: Supplementary file 3 — Additional file 3. Table S1: Baseline characteristics of the propensity score-matched cohort grouped by the medians of the P wave duration and the PR segment. [file 12872_2022_3028_MOESM3_ESM.pdf]

Full title: The Prognostic Predictive Value of the Components of the PR Interval in Hospitalized Patients with Heart Failure

Journal name: *BMC Cardiovascular Disorders*

Authors: Yi-Wen Yu, MD[a], Xue-Mei Zhao, PhD[a], Peng-Chao Tian, MS[a], Lang Zhao, MS[a], Yan Huang, MS[a], Qiong Zhou, MB[a], Mei Zhai, MS[a], Yun-Hong Wang, MD[a], Yu-Hui Zhang, MD[a], Jian Zhang, MD[a]

Affiliation: a. State Key Laboratory of Cardiovascular Disease, Heart Failure Center, Fuwai Hospital, National Center for Cardiovascular Diseases, Chinese Academy of Medical Sciences and Peking Union Medical College

Corresponding author: Jian Zhang, MD. E-mail address: fwzhangjian62@126.com

Fig S1. Univariable correlation analysis of the P wave duration, the PR segment and other ECG and transthoracic echocardiographic parameters.

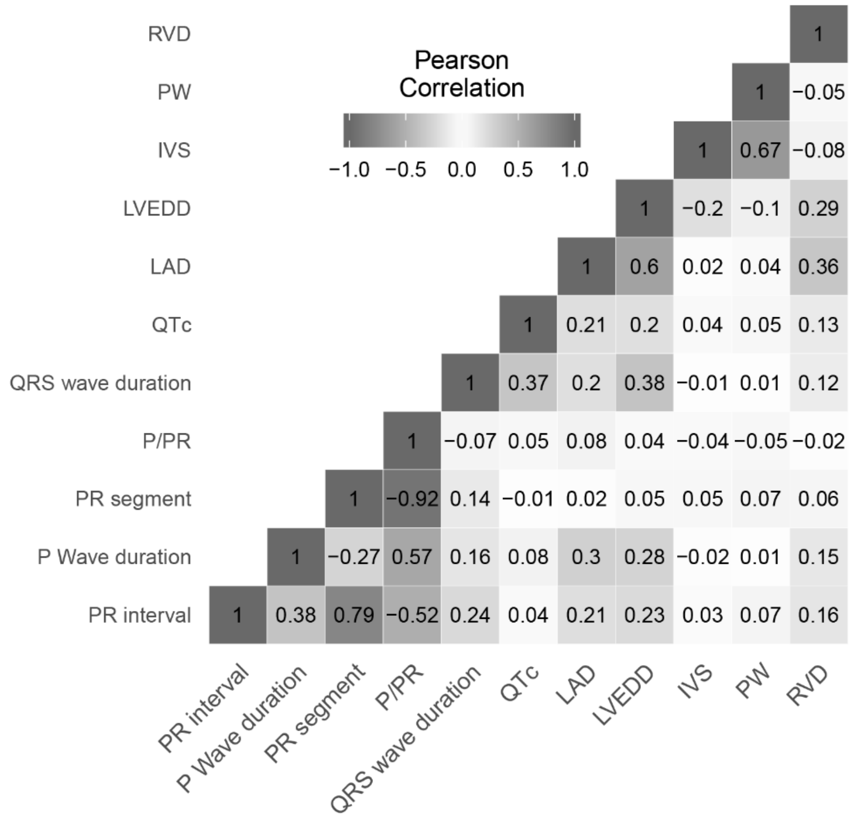

RVD = right ventricular diameter; PW = posterior wall thickness; IVS = interventricular septal thickness; LVEDD = left ventricular end-diastolic diameter; LAD = left atrial diameter; QTc = heart rate corrected QT interval.

Table S1. Baseline characteristics of the propensity score-matched cohort grouped by the medians of the P wave duration and the PR segment

	Shorter P wave duration	Longer P wave duration	*P* value	Shorter PR segment	Longer PR segment	*P* value
Sample size	456	456	/	452	452	/
Age (years)	53.99 (16.09)	53.13 (15.77)	0.413	53.23 (15.94)	54.44 (15.82)	0.254
Male (%)	356 (78.1)	362 (79.4)	0.686	344 (76.1)	347 (76.8)	0.875
Hypertension (%)	25.25 (4.56)	25.40 (4.50)	0.610	25.09 (4.39)	25.21 (4.48)	0.674
SBP (mmHg)	121.46 (20.09)	120.96 (22.47)	0.721	120.26 (20.63)	120.87 (20.38)	0.657
ACEI/ARB (%)	285 (62.5)	285 (62.5)	1.000	286 (63.3)	275 (60.8)	0.493
Hemoglobin (g/L)	141.02 (21.10)	142.59 (21.86)	0.271	139.95 (22.48)	140.06 (22.71)	0.945
Serum albumin (g/L)	40.39 (5.27)	40.25 (5.22)	0.686	40.18 (5.27)	40.05 (5.23)	0.723
Serum potassium (mmol/L)	4.00 (0.46)	3.99 (0.47)	0.791	4.00 (0.46)	4.01 (0.50)	0.819
eGFR (ml/min/1.73m^2)	72.81 (24.72)	73.21 (23.84)	0.805	73.97 (24.84)	73.16 (25.06)	0.629
Heart rate (bpm)	78.47 (16.24)	78.71 (14.63)	0.813	78.17 (15.37)	78.22 (14.69)	0.964

QRS complex duration (ms)	109.84 (25.96)	110.93 (23.90)	0.512	109.02 (24.51)	109.86 (25.85)	0.619

The continuous variables were expressed as the mean (standard deviation), and categorical variables were expressed as the frequency (percentage). Shorter and longer respectively meant less than or equal to the median of the research variables and greater than the median.

Fig S2. The distribution of the standardized mean differences before and after propensity score matching. A. P wave duration; B. PR segment.

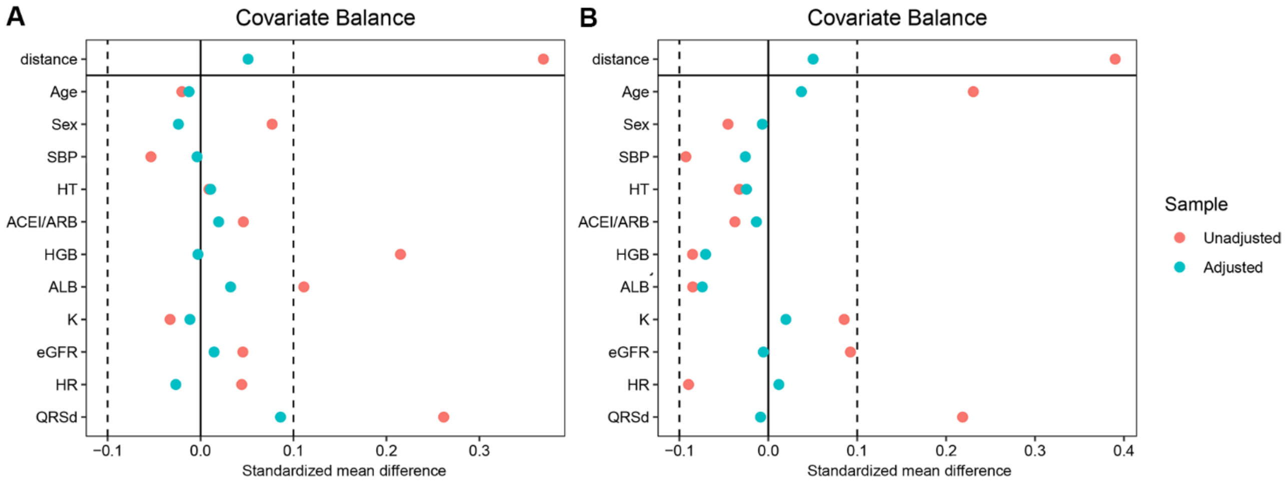

SBP = systolic blood pressure; HT = hypertension; ACEI/ARB = angiotensin-converting enzyme inhibitors/angiotensin-receptor blockers; HGB = hemoglobin; ALB = albumin; K = potassium; eGFR = estimated glomerular filtration rate; HR = heart rate; QRSd = QRS complex duration.
